# A New Benzofuran Derivative from *Flemingia philippinensis* Merr. et Rolfe

**DOI:** 10.3390/molecules17077637

**Published:** 2012-06-25

**Authors:** Hua Li, Fengyan Zhai, Meihua Yang, Xiaowan Li, Pingli Wang, Xiaojun Ma

**Affiliations:** 1 Institute of Medicinal Plant Development, Chinese Academy of Medical Sciences and Peking Union Medical College, Beijing 100094, China; 2 Department of Food Science, Henan University of Technology, Zhengzhou 450001, Henan, China; 3 Department of Resource and Environment, Henan Institute of Science and Technology, Xinxiang 453003, Henan, China; 4 Biology Technology Institute, Xinjiang Agricultural Vocational and Technical Institute, Changji 831100, Xinjiang, China; 5 Guangxi Botanical Garden of Medicinal Plant, Nanning 530023, Guangxi, China

**Keywords:** *Flemingia philippinensis*, flemiphilippinone A, flavonoids

## Abstract

A new prenylated benzofuran derivative, named flemiphilippinone A, was isolated together with ten known flavonoids from the roots of *Flemingia philippinensis*. Flemiphilippinone A was identified as (2*S*,3a*S*)-5-(1-hydroxy-3-(4-methoxyphenyl)- propylidene)-2-(2-hydroxypropan-2-yl)-3a,7-bis(3-methylbut-2-en-1-yl)tetrahydrobenzo- furan-4,6(2*H*,5*H*)-dione, and its structure was established by a combination of HR EIMS, ^1^H-NMR, ^13^C-NMR, HMQC, HMBC and NOESY spectra data.

## 1. Introduction

**Figure 1:**
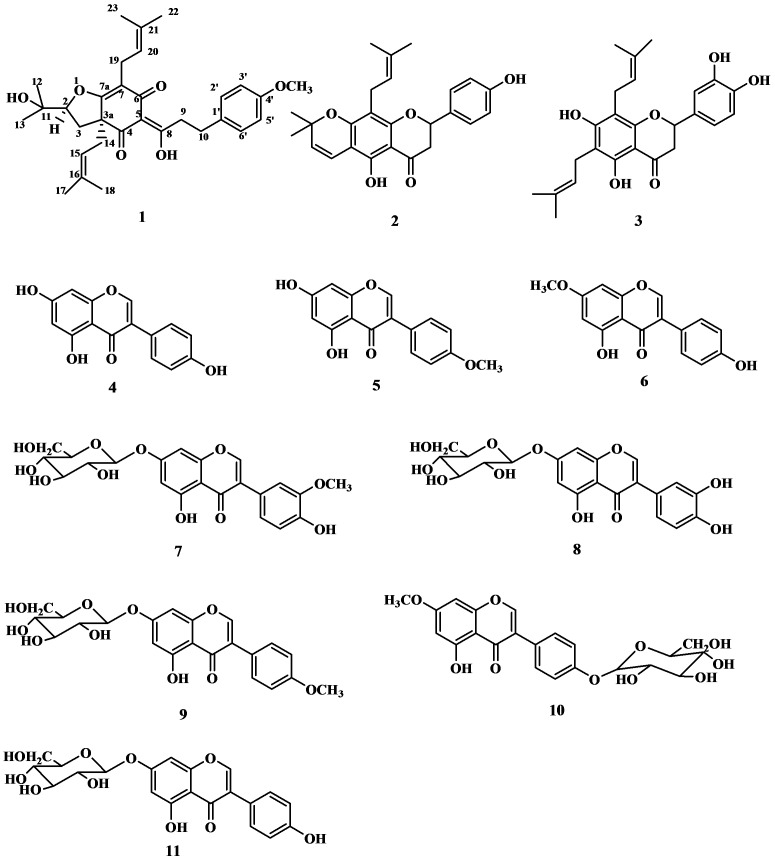
The chemical structures of compounds **1–11**.

Early studies regarding the chemical constituents of *Flemingia philippinensis* have revealed the presence of flavonoids, steroids and triterpenes [1,2,3]. In our previous research work on *F. philippinensis*, flavonoids, flavonoid glycosides, anthraquinones and organic acids were isolated [4,5,6]. In continuation of our research on the medicinal plant *F. philippinensis*, we have performed a phytochemical investigation on the root of *F. philippinensis*, which has led to the isolation of a new compound named flemiphilippinone A (**1**). In addition, ten known flavonoids were identified as lupinifolin (**2**) [7], 6,8-diprenyleriodictyol (**3**) [8], genistein (**4**) [9], biochanin A (**5**) [10], prunetin (**6**) [11], 3′-*O*-methylorobol-7-*O*-glycoside (**7**) [12], luteoloside (**8**) [13], sissotrin (**9**) [14], prunetin 4′-*O*-glycoside (**10**) [15] and genistin (**11**) [15] on the basis of their UV, IR, EI MS, ^1^H-NMR, ^13^C-NMR spectral data and by comparison of spectral values reported in the literature. All compounds (the chemical structures shown in Figure 1), except **4**, **5** and **11**, were isolated from *F. philippinensis* at the first time and compound **1** was a new compound.

## 2. Results and Discussion

**Figure 2:**
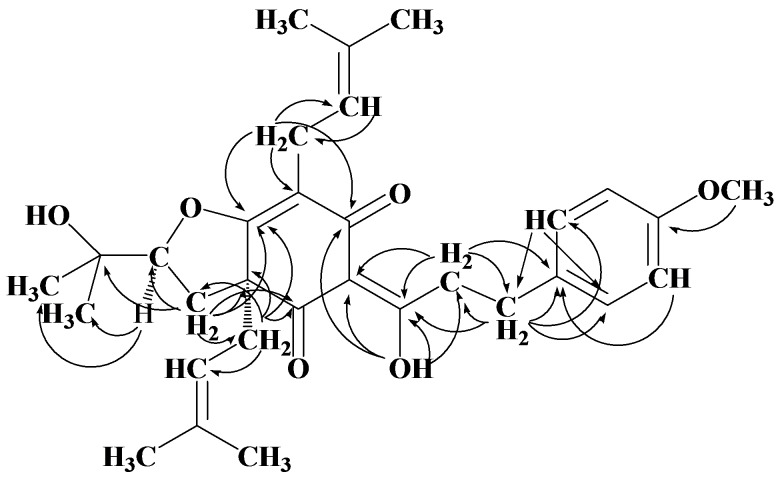
Selected HMBC (H-C) of compound **1**.

Flemiphilippinone A (**1**) was isolated as a light yellow oily liquid. The HR-EI-MS revealed a pseudomolecular ion peak at *m/z* 508.2788 [M]^+^, thus suggesting a molecular formula of C_31_H_40_O_6_ (calc. 508.2825). The ^1^H-NMR spectrum (Table 1) established the presence of four aromatic protons (*δ* 7.19 and 6.82). Two doublet proton signals at *δ* 7.19 (2H, d, *J* = 8.5 Hz, H-2′, 6′) and *δ* 6.82 (2H, d, *J* = 8.5 Hz, H-3′, 5′) were correlated with ^13^C signals at 130.4 (C-2′, 6′) and 115.6 (C-3′, 5′), respectively, in the HMQC spectrum, indicating the presence of an AA′BB′ system in a benzene ring. The signal at *δ* 3.77 (3H, s) was correlated to the carbon signal at *δ* 158.0 (C-4′) in HMBC spectrum (shown in Figure 2) and correlated to H-3′, 5′ in NOE spectrum, revealed a methoxy group was substituted at C-4′ of benzene ring. Two protons at δ 2.92 (1H, m) and δ 2.85 (1H, m) related with δ 31.0 (C-10) shown in HMQC spectrum, and which related with δ 198.2 (C-8), 40.0 (C-9), 133.3 (C-1′) and 113.7 (C-2′, 6′), suggesting that a 4-methoxy-substituted phenmethyl group was present. What’s more, two protons on the methylene group (C-9) correlated to δ 31.0 (C-10) and 133.3 (C-1′), respectively, in the HMBC spectrum, indicating that the compound contained a 4-methoxy-substituted phenylethyl fragment, corresponding with the molecular weight of 121 and 135 in mass spectrum. In the HMBC spectrum, the signal at *δ* 18.84 (-OH, s) was correlated with the carbon signals at *δ* 106.5 (C-7), 192.3 (C-6), 198.2 (C-8) and 40.0 (C-9), indicating that the hydroxyl at *δ* 18.84 at low magnetic field connected with a six-membered ring, neighboring two adjacent ketones, and substituted at C-8, which is a structure similar to that of the prenylated benzophenone compound garciniaone [16,17]. What more, C-9 was correlated to C-8 according to the relations between H-9 and other carbons.

In the structure of this compound, two 3-methylbut-2-en-1-yl groups were was identified by the following ^1^H-NMR signals at *δ* 3.10 (1H, m, H-19), 3.00 (1H, dd, *J* = 7.5, 14.5 Hz, H-19), 5.11 (1H, t, *J* = 7.5 Hz, H-20), 1.72 (3H, s, H-22) and 1.68 (3H, s, H-23), and corresponding ^13^C signals at *δ* 21.5 (C-19), 121.5 (C-20), 17.8 (C-22), 17.8 (C-23), respectively, assigned by the HMQC spectrum. The HMBC data indicated that C-7 was substituted by a 3-methylbut-2-en-1-yl group due to the correlation between H-19 and *δ* 107.3 (C-5), 192.3 (C-6) and 175.1 (C-4). The presence of another 3-methylbut-2-en-1-yl group was confirmed by the ^1^H-NMR signals at *δ* 2.50 (1H, dd, *J* =8.0, 14.0 Hz, H-14), 2.39 (1H, dd, *J* = 7.5, 14.0 Hz, H-14), 4.96 (1H, t, *J* = 7.5 Hz, H-15), 1.66 (3H, s, H-17) and 1.52 (3H, s, H-18). According to the correlation between H-15 and *δ* 60.6 (C-3a), 175.1 (C-4) and 195.7 (C-7a), C-3a was substituted by the prenyl group. All the assignments of ^1^H- and ^13^C-NMR data for the compound were achieved by HMQC and HMBC experiments.

The signal *δ* 4.51 (1H, dd, *J* = 6.5, 10.0 Hz, H-2) was split into a dd peak by 2.16 (2H, m, H-3) which correlated to *δ* 90.5 (C-2), 60.6 (C-3a), 175.1 (C-4), 195.7 (C-7a), 71.2 (C-11) and 38.7 (C-14) in HMBC spectrum, indicating that C-2 connected with C-3a through C-3. Two single signals *δ* 1.33 and 1.16 at C-11, were correlated to *δ* 71.8 (C-11) and 90.5 (C-2). Moreover, C-7a (*δ* 195.7) was linked to C-7 (*δ* 106.5) by double bond and C-7a connected with C-5 (*δ* 90.5) through an oxygen bridge. Beside the partial structures mentioned above, the molecular formula C_31_H_40_O_6_ requires a hydroxyl group which must be connected with C-11, which was a quaternary carbon and chemical shift appeared at 71.8 in the low magnetic field.

**Table 1:** ^1^H (500 MHz), ^13^C (125 MHz) and 2D-NMR (500 MHz) data of **1** in CDCl_3_.

Position	*δ*_H_ *J*(Hz)	*δ*_C_	HMBC	COSY	NOE
2	4.51 dd (6.5, 10)	90.5	C-12, 13	H-3	H-3, 12, 13, 14
3	2.16 m	30.6	C-2,3a, 4, 7a, 11, 14	H-2	H-13
3a		60.6			
4		175.1			
5		107.3			
6		192.3			
7		106.5			
7a		195.7			
8(-OH)	18.84 s	198.2	C-6, 7, 8, 9		
9	3.18 m	40.0	C-7, 8, 10, 1′		
	3.10 m				
10	2.92 m	31.0	C-8, 9, 1′, 2′, 6′		
	2.85 m				
1′		133.3			
2′	7.19 d (8.5)	113.7	C-10, 3′, 4′, 6′		
3′	6.82 d (8.5)	129.4	C-1′, 4′, 5′		OCH_3_-4′
4′		158.0			
5′	6.82 d (8.5)	129.4	C-1′, 3′, 4′		OCH_3_-4′
6′	7.19 d (8.5)	113.8	C-10, 2′, 4′, 5′		
11		71.2			
12	1.33 s	26.6	C-2, 11, 13		H-2
13	1.16 s	23.8	C-2, 11, 12		H-2
14	2.50 dd (7.5, 14.0)	38.7	C-3a, 4, 7a, 3, 15, 16	H-15	H-2
	2.39 dd (7.5, 14.0)				
15	4.96 t (7.5)	117.1	C-17, 18	H-14, 17, 18	H-17
16		137.0			
17	1.66 s	25.8	C-15, 16, 18	H-15	H-15
18	1.52 s	17.8	C-15, 16, 17	H-15	
19	3.10 m	21.5	C-4, 5, 6, 20, 21	H-20, 23	
	3.00 dd (7.5, 14.5)				
20	5.11 t (7.5)	121.5	C-19, 22, 23	H-19, 23	H-23
21		132.1			
22	1.72 s	17.8	C-20, 21, 23		
23	1.68 s	25.6	C-20, 21, 22	H-19, 20	
4′(-OCH_3_)	3.77 s	55.2	C-4′		H-3′, 5′

The stereochemistry of the proton at C-2 and butenyl group at C-3a in the structure was determined by the H-H correlations in the NOESY spectrum. The configurations of proton at C-2 was determined by the correlations of H-C (2), Me (12), Me (13), and H-C (14) which were assigned to be in an orientation on ground. According to the size of the connection groups at C-2, the stereochemistry was an *S*-configuration. In addition, the ROESY correlations of H-C (7) with H-C (19) and H-C (20), and H-7 (21) with H-C (21) were assigned, indicating the stereochemistry of butenyl group at C-3a was also in an *S*-configuration. Therefore, the structure of flemiphilippinone A (**1**) was determined to be (2*S*,3a*S*)-5-(1-hydroxy-3-(4-methoxyphenyl)propylidene)-2-(2-hydroxypropan-2-yl)-3a,7-bis-(3-methylbut-2-en-1-yl)tetrahydrobenzofuran-4,6(2*H*,5*H*)-dione.

## 3. Experimental

### 3.1. General

TLC was preformed with silica gel GF254 (Marine Chemical Industry Factory, Qingdao, China), and the spots were visualized by spraying with 10% H_2_SO_4_-EtOH reagent, followed by heating. Column chromatography was performed using silica gel (Qingdao Haiyang Chemical Co., Ltd, Qingdao, China), reverse-phase C18 silica gel (50 μm, Merck, city Germany) and Sephadex LH-20 (Amersham Pharmacia Biotech, Uppsala, Sweden). All reagents were analytical grade and water was distilled-twice. Optical rotation was measured on Perkin-Elmer 341 polarimeter (Fremont, CA, USA). UV spectra were measured with a Shimadzu UV-2550 visible spectrophotometer (Shimadzu, Japan). IR spectra were recorded on a NEXUS-470 FTIR (Nicolet, Jakarta Raya, Indonesia). All NMR experiments were performed on a Bruker DRX-500 spectrometer (Bruker, Switzerland) (500 MHz for 1H and 125 MHz for 13C) equipped with 5 mm probe head (PADUL 13C). The chemical shifts were with 0.03% tetramethylsilane as an internal reference. About 3–10 mg samples were dissolved in CDCl3 or CD3OD (0.5 mL) to record the NMR spectra. EIMS and HR EIMS spectra were taken on an Auto Spec-ultima mass spectrometer (VG Co., English) at an ionization voltage of 70 eV.

### 3.2. Plant Material

The fresh roots were collected from Guangxi Botanical Garden of Medicinal Plant, Nanning, China in April 2007. A voucher specimen has been deposited in the Herbarium of the Institute of Medicinal Plant Development, Chinese Academy of Medical Science and Peking Union Medical College.

### 3.3. Extraction and Isolation of Chemical Constituents

The roots of *F. philippinensis* (30 kg) were dried, powdered and extracted three times with 75% EtOH (solid to liquid ratio 1:10, 24 h, room temperature 20 °C). After removal of the solvent by evaporation, the residue was suspended in water and defatted with petroleum ether. Then the aqueous layer was dried to afford the residue (723 g), which was subjected to the silica gel (200–300 mesh, 2 kg) column chromatography using CHCl_3_-MeOH step-gradient elution (1:0–20:1–10:1–5:1–0:1, v/v) to yield five fractions (B1, B2, B3, B4 and B5). The fraction B1 (28 g) was subjected to a silica gel (200–300 mesh, 350 g) column chromatography (CC) again using petroleum ether-acetone (P-EA, from 20:1 to 0:1). The fraction eluted with P-EA (20:1) was subjected to silica gel chromatography (eluted with CHCl_3_: MeOH, from 1:0 to 5:1) and the part eluted by 20:1 was further separated by ODS-silica gel chromatography (eluted with 70–90% MeOH) and obtained the compound **1** (17 mg). One of fractions eluted with P-EA (15:1) was subjected to silica gel chromatography (eluted with CHCl_3_/MeOH = 20:1) and Sephadex LH-20 CC to yield one compound and two fractions which were further purified repeatedly by ODS-silica gel chromatography (eluted with 70–90% MeOH) to afford compound **2** (3 mg) and **3** (0.4 mg), respectively. The fraction B2 (18 g) was subjected to silica gel (200–300 mesh, 300 g) CC again using CHCl_3_-MeOH step-gradient elution (1:0–20:1–10:1) and the mixture of **5** and **6** (31 mg) was obtained from the third part eluted with 10:1 through further separation using Sephadex LH-20 and ODS-silica gel (eluted with 70–80% MeOH) CC. The fraction B3 (12 g) was also subjected to a silica gel (200–300 mesh, 300 g) CC using CHCl_3_-MeOH step-gradient elution (1:0–5:1). Compound **7** (7 mg), **8** (6 mg) and the mixture **9** and **10** (23 mg) were obtained from the two fractions eluted with 20:1 and 10:1 by repeated CC on silica gel, followed by Sephadex LH-20 and ODS-silica gel (70% MeOH). At the same time, compound **4** (102 mg) and **11** (120 mg) were also obtained during the course of separation of the above compounds from fractions B2 and B3, respectively. The solvent system CHCl_3_-MeOH (2:3) was used as the eluent on Sephadex LH-20 CC in the whole experiment.

### 3.4. Flemiphilippinone A *(**1**)*

Light yellow oily liquid (CHCl_3_-MeOH); [α]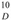: +0.050° (c 0.10, CHCl_3_); UV (MeOH) λ max (log εmax): 277 (0.59), 223 (1.40) nm. IR (KBr) ν max: 3455, 2927, 2971, 1669, 1579, 1512, 1453, 1375, 1245, 1180, 1107, 1071, 1035, 826 cm^−1^. ^1^H- and ^13^C-NMR: see Table 1. EI MS *m*/*z*: 508 [M] ^+^ (13), 439 (100), 383 (15), 305 (31), 278 (11), 249 (29), 177 (13), 121 (100), 69 (48). HR EIMS *m*/*z*: 508.2788 (calc. for C_31_H_40_O_6_, 508.2825).

## 4. Conclusions

Repeated column chromatography (including normal-phase silica gel, RP-silica gel and Sephadex LH-20) of the EtOH extract of the roots of *F. philippinensis* has led to the isolation of flemiphilippinone A (**1**), together with ten known flavonoids, including two prenylated flavanones, three simple isoflavones and five isoflavone glycosides, which were identified as lupinifolin (**2**), 6,8-diprenyleriodictyol (**3**), genistein (**4**), biochanin A (**5**), prunetin (**6**), 3′-*O*-methylorobol- 7-*O*-glycoside (**7**), luteoloside (**8**), sissotrin (**9**), prunetin 4′-*O*-glycoside (**10**) and genistin (**11**). The structure of the new compound **1** was determined by spectroscopic methods, including 1D NMR, 2D NMR and MS experiments, and the structures of the known compounds including **2~11** were identified mainly by comparing their NMR data with those in the literature. In addition, it is worth mention that isoflavones and flavanones as two types of flavonoid compounds that could be considered chemomarkers of *F. philippinensis* at a higher level within species of the genus *Flemingia*. Flemiphilippinone A, was a novel compound, was firstly isolated from the Leguminosae, which potent activity and correlation with the antioxidant activity of *F. philippinensis* should be investigated further.

## Supplementary Materials

Supplementary materials can be accessed at: http://www.mdpi.com/1420-3049/17/7/7637/s1.
