# Supplementary material for: A New Benzofuran Derivative from Flemingia philippinensis Merr. et Rolfe
Source: Molecules. 2012 Jun 25;17(7):7637–44. doi: 10.3390/molecules17077637 (PMC6268854; doi:10.3390/molecules17077637)
Supplement: Supplementary file 1 [file molecules-17-07637-s001.pdf]

Supplementary Materials

NMR data of compounds 2–11

Table 1. ^{13}C-NMR (125 MHz) and ^{1}H-NMR (500 MHz) data of **2**, **3** in CDC1$_3$.

Position	2		3	
	C	H	C	H
2	78.5	5.33 dd (3.0, 12.5)	79.5	5.33 dd (3.0, 13)
3	43.0	2.80 dd (3.0, 17.0),<br>3.03 dd (12.5, 17.0)	44.2	2.79 dd (3.0, 17.0),<br>3.05 dd (12.5, 17.0)
4	196.8		197.8	
5	156.5		160.4	
6	108.7		108.5	
7	160.1		162.8	
8	102.8		106.5	
9	159.4		158.8	
10	102.6		103.7	
1′	130.6		131.7	
2′	127.6	7.32 dd (3.0, 8.0)	113.8	7.20 d (2.5)
3′	115.5	6.87 dd (3.0, 8.0)	144.8	
4′	156.1		145.6	
5′	115.5	6.87 dd (3.0, 8.0)	116.2	6.84 d (8.0)
6′	127.6	7.32 dd (3.0, 8.0)	119.3	7.20 dd (2.0, 8.0)
1″	115.5	6.64 d (10.0)	21.9	3.31 d (7.0)
2″	126.0	5.50 d (10.0)	122.0	5.22 t (7.0)
3″	78.2		134.9	
4″	28.3	1.45 s	17.9	1.73 s
5″	28.3	1.45 s	25.8	1.73 s
1‴	21.4	3.20 d (7.0)	21.3	3.38 d (7.0)
2‴	122.4	5.15 t (7.0)	121.6	5.33 t (7.0)
3‴	131.1		134.3	
4‴	17.8	1.45 s	17.9	1.79 s
5‴	25.7	1.47 s	25.8	1.79 s
-OH		12.24 br s		5.97 br s; 6.02 br s

Table 2. ^{13}C-NMR (125 MHz) and ^{1}H-NMR (500 MHz) data of **4**, **5** and **6** in in CD_3OD.

Position	4		5		6	
	H	C	H	C	H	C
2	8.03 s	155.3	8.09 s	155.6	8.06 s	155.5
3		125.3		132.5		130.5
4		182.8		181.0		181.0
5		164.4		166.5		161.5
6	6.20 d (1.0)	100.6	6.34 d (2.5)	99.7	6.21 d (2.5)	100.7
7		166.4		168.0		166.0
8	6.33 d (1.0)	95.3	6.51 d (2.5)	93.8	6.32 d (2.5)	95.3
9		160.2		164.3		161.5
10		106.8		117.5		116.5
1′		123.8		125.0		125.0
2′	7.36 dd (2.0, 7.0)	131.9	7.37 d (9.0)	131.9	7.46 d (9.0)	131.9
3′	6.83 dd (2.0, 7.0)	116.8	6.84 d (9.0)	116.8	6.84 d (9.0)	115.4
4′		159.3		169.1		159.0
5′	6.83 dd (2.0, 7.0)	116.8	6.84 d (9.0)	116.8	6.84 d (9.0)	115.4
6′	7.36 dd (2.0, 7.0)	131.9	7.37 d (9.0)	131.9	7.46 d (9.0)	131.9
$-OCH_3$			3.87 s	56.97	3.82 s	56.26

Table 3. ^{1}H-NMR (500 MHz) and ^{13}C-NMR (125 MHz) data of **7**, **8**, **9**, **10** and **11**.

Position	7 (CD_3OD)		8 (CD_3OD)		9 (CD_3OD)		10 (CD_3OD)		11 (DMSO)	
	H	C	H	C	H	C	H	C	H	C
2	8.14	155.7	8.13 s	154.8	8.14 s	154.0	8.14 s	154.1	8.42 s	154.8
3		125.2		124.7		123.2		124.9		122.7
4		182.6		180.6		180.9		181.0		180.6
5		163.7		162.8		162.2		162.3		161.8
6	6.49 d (2.5)	101.3	6.51 d (2.0)	100.0	6.52 d (2.0)	100.9	6.35 d (2.0)	100.3	6.46 d (1.5)	100.1
7		164.9		164.2		166.0		163.4		163.2
8	6.67 d (2.0)	96.1	6.69 d (2.0)	94.7	6.69 d (2.0)	94.5	6.50 d (2.0)	92.0	6.82 d (1.5)	94.7
9		159.4		157.7		158.2		159.9		157.6
10		108.2		106.8		106.8		105.7		106.2
1′		114.1		122.1		123.0		123.4		121.1
2′	7.16 d (1.0)	123.1	7.06 d (1.5)	116.2	7.48 d (8.5)	129.9	7.48 d (9.0)	129.9	7.38 d (8.0)	130.3
3′		149.0		150.6	6.97 d (9.0)	113.5	6.98 d (9.0)	116.4	6.82 d (8.0)	115.2
4′		148.2		157.4		157.9		157.8		157.4
5′	6.84 d (8.5)	123.7	6.86 d (8.0)	116.2	6.97 d (9.0)	113.5	6.98 d (9.0)	116.4	6.82 d (8.0)	115.2
6′	6.96 dd (1.5, 8.0)	116.4	6.99 dd (2.0, 8.0)	130.6	7.48 d (8.5)	129.9	7.48 d (9.0)	129.9	7.38 d (8.0)	130.3
1″	5.02 d (7.5)	101.8	5.07 t (6.5)	100.7	5.03 t (7.5)	99.8	4.95 t (7.5)	97.9	5.06 d (7.5)	99.7
2″		74.9		73.7		73.3		73.6		73.2
3″		78.5		77.5		76.8		77.0		77.3
4″		71.4		70.0		69.9		70.0		69.8
5″		78.0		76.9		76.5		76.6		76.6
6″		62.6		61.8		61.1		61.2		60.8
2″~6″	3.38~3.91		3.40~4.00		3.30~4.00		3.30~3.91		3.10–3.80	
-OH					3.82 s	54.4	3.87 s	55.1	12.92 br s; 9.53 br s	
$-OCH_3$	3.88	56.7			8.14 s	154.0	8.14 s	154.1		
